# Comparative Evaluation of the Performance of a Mobile Device Camera and a Full-Frame Mirrorless Camera in Close-Range Photogrammetry Applications

**DOI:** 10.3390/s24154925

**Published:** 2024-07-30

**Authors:** Photis Patonis

**Affiliations:** School of Rural & Surveying Engineering, Aristotle University of Thessaloniki, University Box 439, GR-54 124 Thessaloniki, Greece; patonis@auth.gr; Tel.: +30-231-099-4207

**Keywords:** photogrammetry, smartphones, full-frame mirrorless camera, distortions, accuracy, image quality

## Abstract

The comparative evaluation of the performance of a mobile device camera and an affordable full-frame mirrorless camera in close-range photogrammetry applications involves assessing the capabilities of these two types of cameras in capturing images for 3D measurement purposes. In this study, experiments are conducted to compare the distortion levels, the accuracy performance, and the image quality of a mobile device camera against a full-frame mirrorless camera when used in close-range photogrammetry applications in various settings. Analytical methodologies and specialized digital tools are used to evaluate the results. In the end, generalized conclusions are drawn for using each technology in close-range photogrammetry applications.

## 1. Introduction

Photogrammetry primarily concerns precise three-dimensional measurements of objects and terrain features from photographs. It can be used in fields such as topographic mapping [1], architecture [2], archaeology [3], filmmaking [4], engineering [5], manufacturing [6], quality control [7], police investigation [8], cultural heritage [9], underwater site mapping [10], and geology [11]. Photogrammetry has numerous advantages over other surveying methods, such as accuracy, low cost, and speed. In photogrammetry, the selection of cameras depends on project requirements and budget. The most crucial criterion about the requirements is the accuracy achieved in the results, which depends on the final mapping product’s scale.

**Figure 1:**
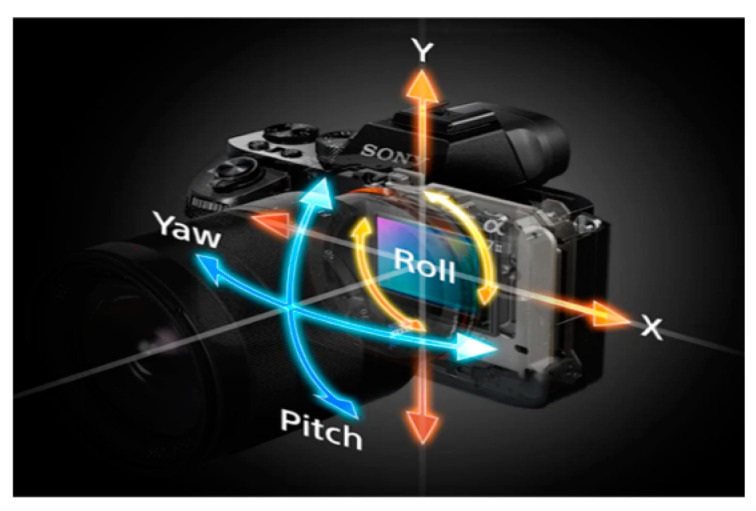
Sensor-based, image stabilization system [12].

Full-frame mirrorless cameras are the most advanced still cameras available on the market today. Full-frame cameras are equipped with an image sensor that is the size of a classic 35 mm analog film frame. The primary difference between a full frame and a crop sensor is the physical size of the sensor; crop sensors are smaller and can vary in dimensions. Mirrorless cameras offer several advantages over Digital Single-Lens Reflex (DSLR) cameras, including the compact size and faster shooting speed. Some of the most sophisticated still cameras have a 5-axis, sensor-based, image stabilization system that can move or rotate the image sensor along five different axes to counteract camera shake, see Figure 1.

Smartphones are designed to be convenient and easy to use, time-saving, and mostly, if not completely, automated. In addition, they are equipped with cameras that capture high-resolution and acceptable-quality photos. The main difference between camera phones and still cameras is the image sensor’s physical size. The number of megapixels on a phone camera may be higher than that of a still camera. However, the sensor’s physical dimensions are smaller. This means that each pixel on a full-frame sensor is bigger and can capture more light, resulting in higher-quality images with less noise when selecting higher ISO values. A mobile phone lens is typically fixed, wide-angle, with no optical zoom. Additionally, the lenses on smartphones are tiny. Therefore, the quality of the photos does not compare to those taken with a high-quality glass lens of a good still camera.

Digital camera technology has reached 61.0 MP levels [13], with a purchase price of 3.7 K. It could be assumed that this would be the ideal equipment for a photogrammetry application. However, the cost is a significant factor in searching for camera alternatives, possibly with lower capabilities but adequate performance. On the contrary, seeing the increased capabilities of smartphones and their additional features (GNSS, inertial sensors, magnetometers), which make them able to be used in more advanced and complex geomatics applications [14,15,16], the question arises whether they can be used in photogrammetry applications.

Different approaches are available in the literature regarding the evaluation and suitability of mirrorless cameras and smartphone technology for photogrammetry applications. In the paper [17], two still cameras, a mirrorless and a DSLR, are compared to investigate the usability of mirrorless cameras for terrestrial photogrammetry applications. The accuracies in the 3D models and the cross sections created are compared. As a result, it has been concluded that mirrorless cameras and point clouds produced using photographs obtained from these cameras can be used for terrestrial photogrammetry projects. In the paper [18], the experimental results show that the geometric and texture data quality produced by smartphone-based photogrammetry produces data that equal or even exceed DSLR-based photogrammetry. The study [19] indicates that smartphones can be utilized directly to acquire on-site photogrammetric data for 3D modeling and measurement extractions for construction management applications. In all the above studies, it is mentioned that other research should be conducted to strengthen the conclusions they formulate.

The scope of this paper is the comparative evaluation of the performance of a mobile device camera and an affordable full-frame mirrorless camera as concerns the distortion levels, accuracy, and image quality that can be achieved in close-range photogrammetry applications. In the end, it must be concluded whether these technologies can be used in photogrammetry and determine which type of camera is more suited to specific tasks. The experimental results and conclusions from this study will contribute to the research on the potentiality of using these technologies.

Regarding the structure of the work, after this introductory section, the methods used to evaluate and compare the cameras are discussed. Next, the experiment results for the cameras’ evaluation and comparison concerning the distortion levels, accuracy performance, and image quality are presented. This is followed by a discussion concerning the analysis and interpretation of the results, as well as conclusions drawn from the present study.

## 2. Methods

To evaluate and compare two cameras and determine whether they are suitable for photogrammetry applications, lens-induced distortions in images must be studied, as well as the accuracy with which point coordinates can be calculated by photogrammetry and the quality of photos from both cameras.

Methodologies and digital tools for camera calibration and evaluation of the results have been developed and implemented in the Surveyor-Photogrammetry software version 6.0 [20]. In this tool collection, two methods are available, an OpenCV function [21] and the photogrammetric bundle adjustment with additional parameters [22], which use the same camera model [23]. A method that utilizes single-image rectification [24] is employed to evaluate the calibration parameters. The camera calibration results are evaluated by an automated process that includes quality checks at various levels of detail. The evaluation includes the “Rect” indicator describing the overall quality, followed by charts and digital images showing the effect of the calibration results on an evaluation image at a control point level. Regarding the number and the shooting angles of the photos that will be used in the calibration, the standard proposed in the specific work [20] can be used.

To directly compare the distortions from the two cameras, a technique must be found that considers the different dimensions of their imaging sensors. Indeed, the concept of normalized pixels was used in this work. Specifically, the current value in pixels is divided by the maximum number of pixels of a reference length, per sensor so that the maximum number of pixels takes the value 1 in both cases. This way, the distortions can be observed in common diagrams, and comparable results can be obtained.

To estimate the accuracy in the photogrammetric calculation of coordinates, with the cameras under consideration, the photogrammetric bundle adjustment method can be used. The method can consider the coordinates of the checkpoints, the internal orientation, and the distortion coefficients as unknowns, thus helping establish whether these parameters affect the coordinate results in a real-world application.

For the quality check and comparison of photos acquired from two cameras, a series of images must be taken, where printed characters and symbols will be depicted in varied sizes. Depending on whether the characters and symbols are recognizable, sharp, or distorted in the images will tell the difference in quality between the two cameras.

For the equipment selection to be compared, the low cost was considered, which in both cases was chosen to be under 1 K EUR. The smartphone camera was selected to have a higher image sensor resolution than the still camera. Moreover, the still camera should be full-frame, mirrorless, and have a lens that can be adjusted to multiple focal lengths. The two devices selected are typical of their categories. The choice of smartphone was the Samsung Galaxy smartphone A52s 5G [25], and the still camera, the Sony α7II [12]. The technical specifications of both cameras are presented in Table 1.

In the smartphone, the highest resolution of the image sensor (64 MP) is used, while the focal length is fixed at 5 mm. The Sony α7 II is an image-stabilized, full-frame, mirrorless camera. Likewise, the highest available resolution (24.3 MP) is used here too, and four distinct nominal focal lengths of the analog lens, i.e., 28, 34, 50, and 70 mm. This defines several cases that need to be considered. These combinations and their output field of view along the sensor’s horizontal and vertical axis are given in Table 2.

It is evident from Table 2, that the Sony-28 mm camera case is closest to the smartphone features. In the other cases, for the results to be comparable, the shooting distance must be varied to cover the same surface area on the object.

## 3. Camera Evaluation and Comparison

### 3.1. Image Distortion Caused by Camera Lens

**Figure 2:**
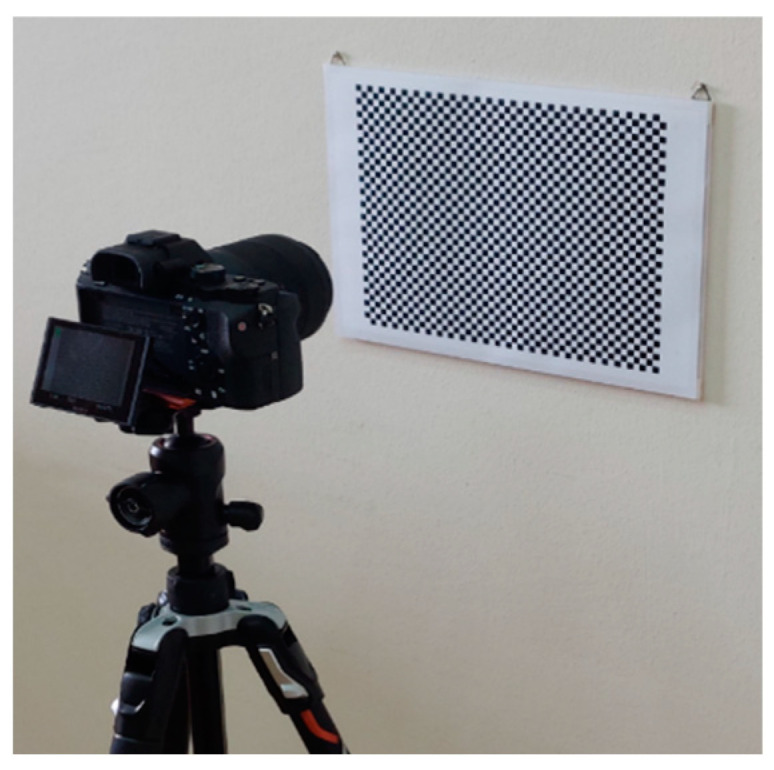
The calibration checkerboard with the Sony α7ΙΙ camera.

Image distortion is a common issue in photography caused by the camera lens producing curved lines where straight lines should be [26]. The two most common types of lens distortion are radial and tangential distortion. Distortion is the result of the lens’s geometrics and can significantly disrupt the image’s quality. The better the quality of a camera’s lens, the smaller the distortions introduced into the image. Especially in photogrammetry, limited distortions are an important factor in obtaining reliable measurement information from images. Alternatively, it is possible to correct the distortions by using mathematical models. In any case, the lack of distortions is better than using these mathematical models, which often roughly describe reality and do not have such precise results. In photogrammetry, lens distortions are calculated using camera calibration, which involves photographing a control field. In this case, a checkerboard with 1813 (49 × 37) control points was utilized, as shown in Figure 2.

The calibration was performed using the Surveyor-Photogrammetry software version 6.0 [20], which, in addition to the estimation of the calibration parameters, also evaluates the results. Two methods were used to determine the magnitude and variation of the distortions in the cameras under review: an OpenCV function and the bundle adjustment method with additional parameters. For the smartphone, five photos were used for the calibration, while for the still camera, seven. That choice was made because the still camera has a narrower sensor than the smartphone camera and requires more photos to centrally cover the entire image sensor. This way, the same checkerboard was used to calibrate both cameras.

An OpenCV function [27] was used to perform the camera calibration, which returns the intrinsic matrix (*f_x_*, *f_y_*, *c_x_*, *c_y_*) and the distortion coefficients matrix (*k*_1_, *k*_2_, *k*_3_, *p*_1_, *p*_2_). The process’s accuracy is described by the total re-projection, the Euclidean distance between the points re-projected using the estimated intrinsic and extrinsic matrix, and the image coordinates of the checkerboard corners. The smaller this error, the better the accuracy of the calculated parameters. The re-projection errors for all experiments are given in Table 3.

The smartphone has a slightly larger re-projection error, which shows a worse performance of the camera calibration parameter values. Whereas for the still camera, it seems in general that as the focal length increases, the parameter application performance worsens.

The intrinsic matrix and the distortion coefficients matrix, as they result from the calibration, are presented in Table 4 and Table 5, respectively. In the intrinsic matrix, the focal length (*f_x_*, *f_y_*), the coordinates of the primary point with respect to the upper left corner of the image (*c_x_*, *c_y_*), the estimation of the aspect ratio *AspectRatio* (*f_y_*/*f_x_*), the nominal focal length *f*, the calibrated focal length *c* in pixels and mm, and the coordinates of the primary point (*x_o_*, *y_o_)* concerning the center of the image in pixels and mm are tabulated.

In Table 4, the *AspectRatio* is approximately 1.000 in all cases, and the calibrated focal length *c* deviates from the nominal focal length *f* from 1.085 mm to 5.341 mm for the still camera and 0.475 mm for the smartphone. In percentages, the still camera’s, focal length differs from the nominal value by 3.9% to 8.2%, while the smartphone’s differs by 9.5%. In any case, the results are realistic.

**Figure 3:**
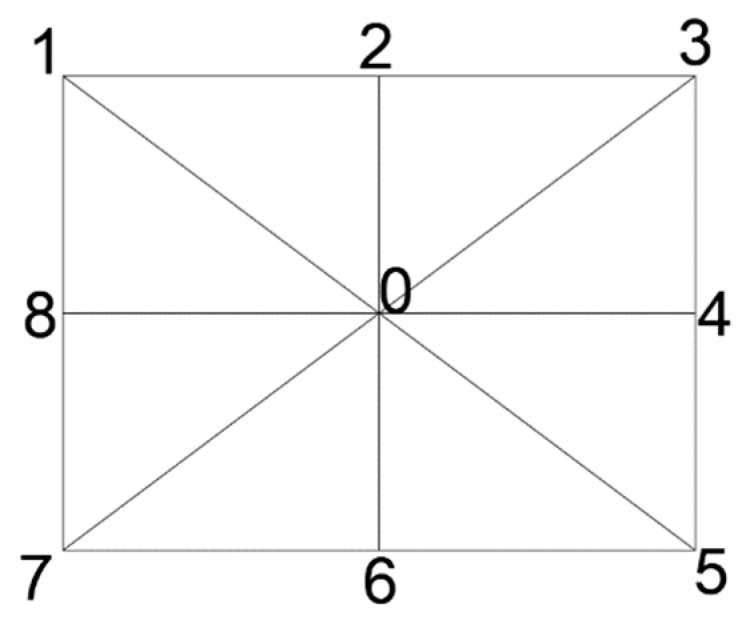
Guide for the evaluation segments.

The coefficients *k*_1_, *k*_2_, *k*_3_, and *p*_1_, *p*_2_, see Table 5, obtained from the calibration process can be applied to the distortion model equations and generate information to provide distortion diagrams. This information concerns the total distortion along pre-defined evaluation segments starting from the primary point and ending at the image’s corners and mid-sides, according to the guide in Figure 3.

**Figure 4:**
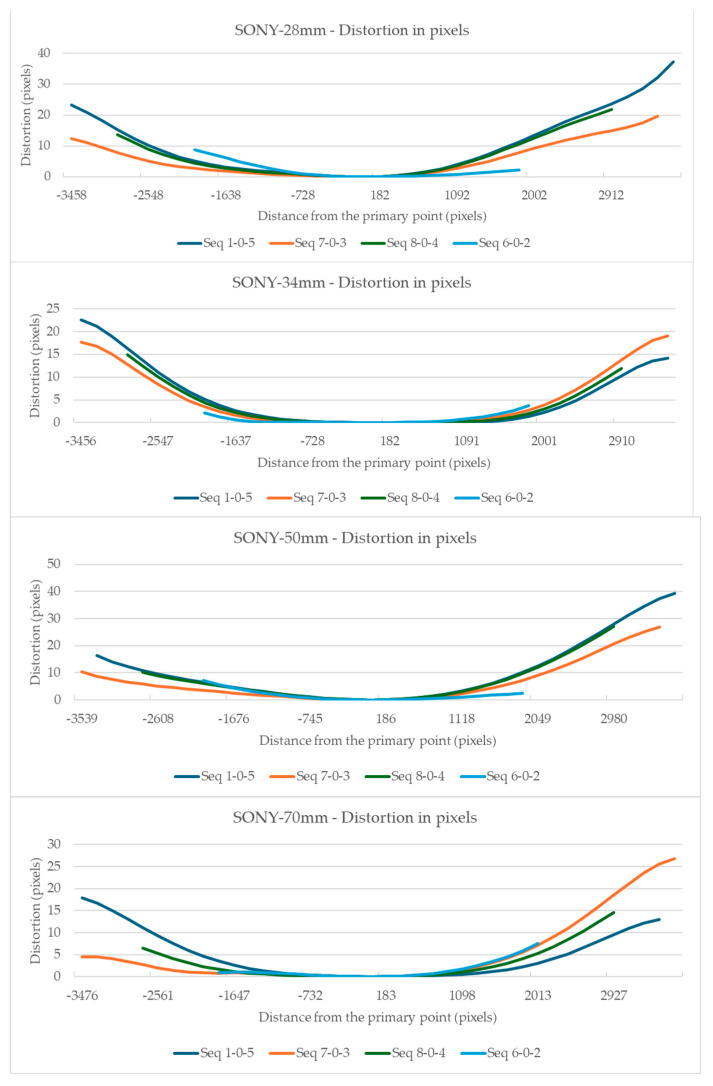
Visualization of the distortion along the evaluation segments for the Sony camera for each focal length.

**Figure 5:**
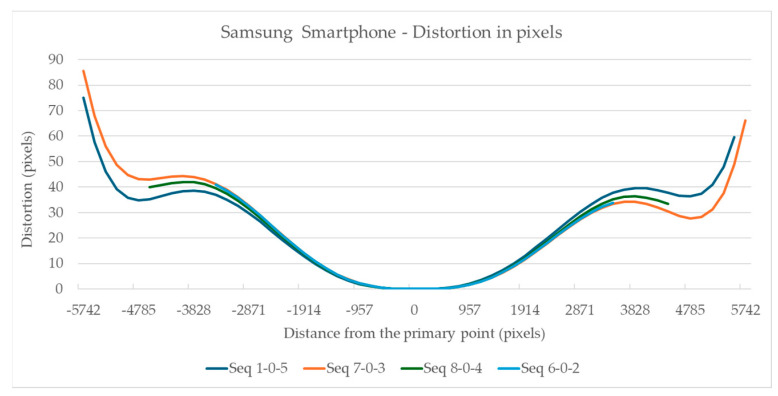
Visualization of the distortion along the evaluation segments for the Samsung smartphone.

The visualization of the total distortion for the evaluation segments 1-0-5, 8-0-4, 7-0-3, and 6-0-2, concerning distance from the primary point, is shown in Figure 4 and Figure 5 for the Sony camera and the Samsung smartphone, respectively.

In the Sony camera’s case, Figure 4 shows a smooth variation in distortion relative to the primary point, at least in the central part of the graphs. No systematic symmetry can be discerned except for the 34 mm focal length, where exceptionally the distortion results show a form of symmetry. 

The total distortion in the Samsung smartphone camera, see Figure 5, shows sharp changes, with the most significant deviations occurring at the ends of the diagonal evaluation segments 7-0-3 and 1-0-5. The form of the chart is symmetric as to the primary point.

**Figure 6:**
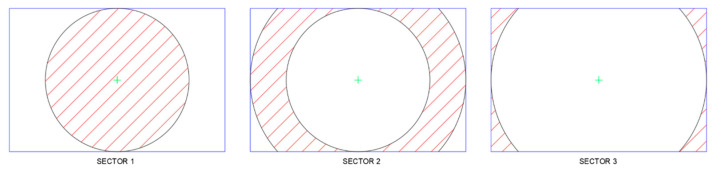
The sensor’s sectors are radially defined from the primary point.

The surface of the sensor was divided into three sectors to generalize the conclusions of the existence of distortion in the sensor areas, see Figure 6. Sector 1 is the circular sector centered at the primary point, which has a radius of the sensor’s height. Sector 2 is defined by the intersection of sector 1 and another circular sector with a radius of the sensor’s width. Finally, Sector 3 is defined as the intersection of the circular sector with a radius of the sensor diagonal and that with a radius of its width.

**Figure 7:**
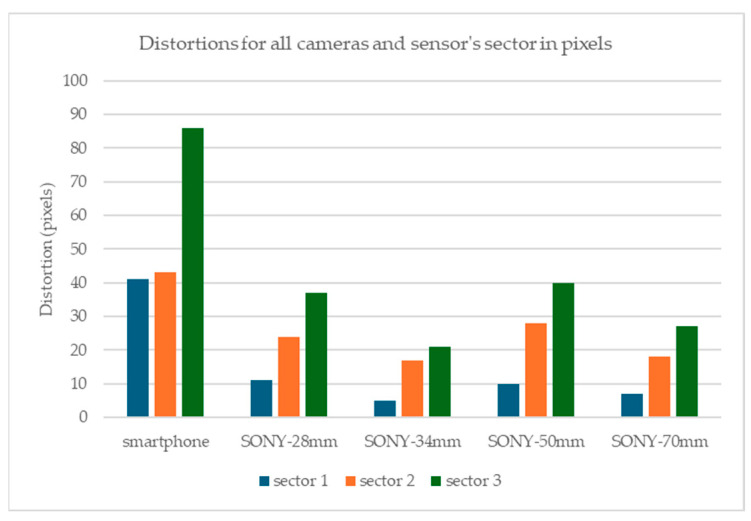
Distortions for all cameras and sensor’s sectors in pixels.

For these sectors, and by examining Figure 4 and Figure 5, the maximum distortions that occurred for the Sony camera and the Samsung smartphone in each sector are displayed in Figure 7.

Figure 7 shows that for the Sony camera, sector 1 has the lowest distortions between 5 and 11 pixels in sector 2, the distortion ranges between 17 and 28 pixels, and in sector 3, 21 and 40 pixels. The best case is the 34 mm lens where the distortions are limited to 5, 17, and 21 pixels for sectors 1, 2, and 3. For the smartphone’s camera, the maximum distortion observed is 41 pixels in sector 1, 43 in sector 2, and 86 in sector 3. 

**Figure 8:**
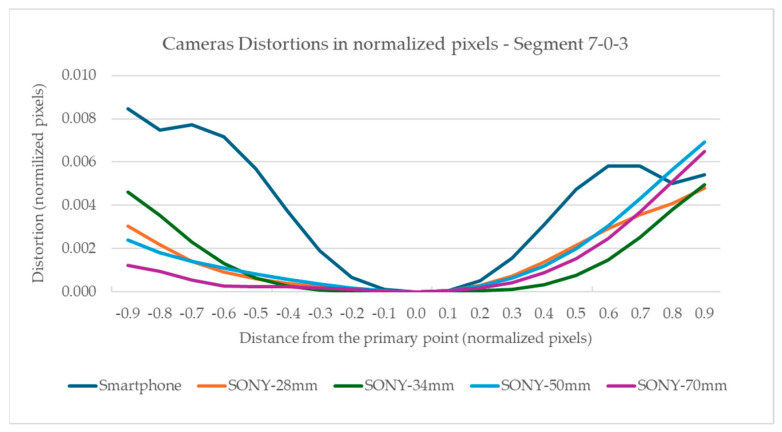
Visualization of the normalized distortions concerning normalized distance from the primary point for all cameras on the 7-0-3 evaluation segment.

**Figure 9:**
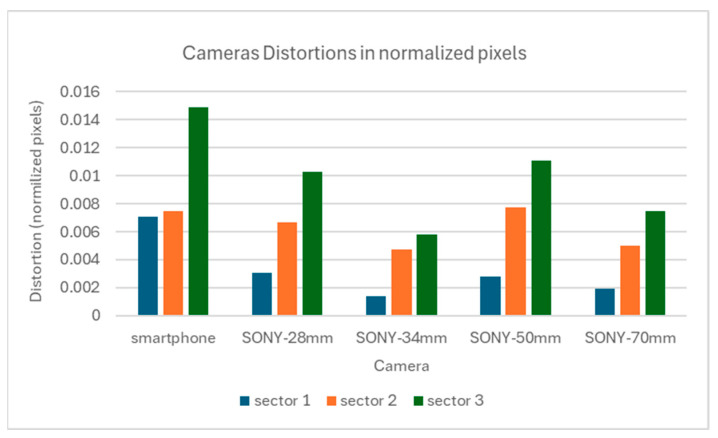
Cameras distortions in normalized pixels in each sector.

To enable a direct comparison of the distortions of the two cameras and their lens combinations, all distortion measurements were normalized to the sensor size. All distortion values and distances from the primary point were divided by the number of pixels along the longest evaluate segments, per camera. The resulting values are shown in Figure 8, for all cameras at the 7-0-3 diagonal evaluation segment and for all cameras, at the sector level, in Figure 9.

Figure 8 shows that the smartphone camera has a more complex form of distortion that increases steeply than the Sony camera distortion. However, the Sony camera presents smoother gradients with characteristic asymmetry towards the primary point. As for the normalized measurement of the total distortion due to lenses, the smartphone camera displays larger values, see Figure 9. However, in the Sony camera, the total distortion is smaller except at the edges of the images; in this case, there is also noticeable distortion.

The alternative for camera calibration is the bundle adjustment method with additional parameters. The method provides more statistics to evaluate the outcomes than the OpenCV function. The general elements of the bundle adjustment solution contain the number of photos, control points used, observations, additional parameters, and degrees of freedom, see Table 6.

In Table 6, the eight additional parameters are the variables *c, x_o_*, *y_o_*, *k*_1_, *k*_2_, *k*_3_, and *p*_1_, *p*_2_. The degrees of freedom are large in both camera calibrations and distinct from each other due to the different number of photos used in each case.

In the analytical solution of the bundle adjustment with additional parameters, the residuals of the unknown parameters indicate the accuracy achieved in the results. The *sigma*, the estimates of the main elements of the internal orientation, and their standard deviations are displayed in Table 7.

In Table 7, the *sigma* ranges at approximately the same levels for all cameras, and the standard deviations in the estimates of the focal length and the coordinates of the primary point are low (1.59–5.82 pixels), except in the case of Sony with the focal length of 70 mm, where the standard deviations are relatively more significant (7.82–11.02 pixels). This order of magnitude of variation in the standard deviations indicates that the results in the camera calibrations are reliable.

The results of the single-image photogrammetry rectification of a photo with the checkerboard are utilized to evaluate the camera calibration parameters [20]. The process is performed twice using the distorted image and the undistorted. The analytical differences between the accuracies in the two cases show the performance of the calibration at a control point level. A positive value means that the calibration parameters have a positive effect, and a negative value means that the calibration parameters do not work effectively. For the visual evaluation of the results, depending on the color gradation, see Table 8, the improvement or worsening of the results is illustrated. Table 9 presents the evaluation images from the application of the internal orientation elements, as derived separately from the OpenCV function and the bundle adjustment method.

From the evaluation images in Table 9, on the Sony camera, depending on the focal length, there appears to be a distinct result each time. In 28, 50, and 70 mm cases, it follows a sort of pattern, but for 34 mm it is a different form. In the Samsung smartphone, it is evident that there is an improvement in most of the image surface, forming a concentric symmetrical shape. The results of applying the distortion coefficients are improved, but without avoiding certain cores where the results are worse, such as Sony’s 28, 34, and 70 mm.

The overall percentage difference between the distorted and undistorted rectifications, called the “Rect” Indicator [20], is an indication of the overall performance of the calibration parameters. The results for each camera are detailed in Table 10.

Using the distortion coefficients to correct the images results in a significant improvement in all cases, see Table 10, with the smartphone camera showing the highest performance rate. The OpenCV method is more effective than the bundle adjustment method in every case, verifying and extending the findings of the study [20]. The results of the photogrammetric bundle adjustment with additional parameters have the same trends but smaller percentages. The same conclusions emerge, in greater detail, from the evaluation images in Table 9 by studying the spatial distribution of the differences.

### 3.2. Accuracy of Geometrical Measurements Extraction

**Figure 10:**
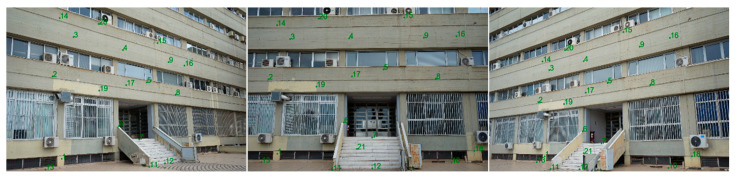
The photos of the test field taken by the Sony-34 mm.

A series of images were taken from each camera to determine the accuracy of extracting geometric measurements, which can be achieved through photogrammetry using the cameras in question. Three converged photographic shots were taken from the smartphone and the still camera for the nominal focal lengths of 28, 34, 50, and 70 mm. Figure 10 shows an example of three photographs depicting the test field taken by the Sony-34 mm camera.

**Figure 11:**
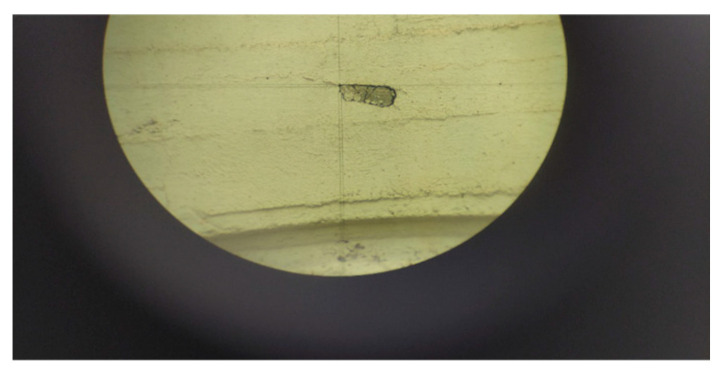
A control point through the telescope of the total station.

For photogrammetric processes to be carried out, points with known ground coordinates must be depicted in the photographs. These points will be used as control points or checkpoints to determine the accuracy achieved in actual three-dimensional measurements. For this purpose, 21 points with known coordinates were used, of which 5 served as checkpoints and the rest as control points. These points are characteristic details on the face of the building, see Figure 11 as an example. To document control points in the field survey, each point code was noted on printed close-up photographs of the object. This allows post-processing to identify the points in the photographs and assign coordinates to them. The coordinates of the points have been measured and calculated by topographic methods [28], which ensure accuracy and reliability [29]. The points were measured with a total station, in reflectorless mode, from the same location. With this technique and considering the measurement distances were close, high precision in calculating the coordinates is ensured, estimated at 2 mm.

**Figure 12:**
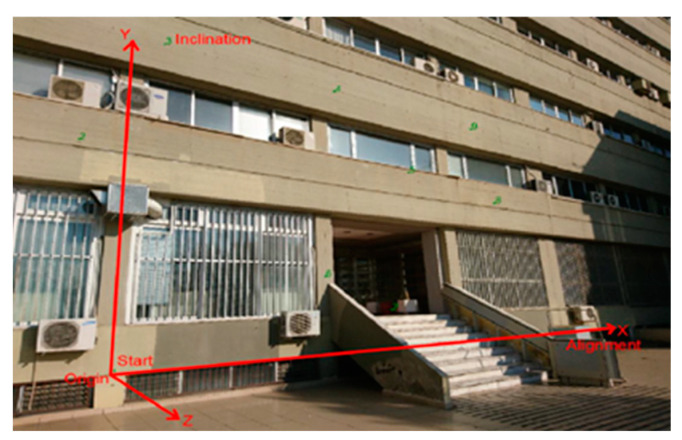
The user coordinate system was adjusted to the facial of the building.

It was considered appropriate to transform the coordinates of the control and checkpoints into a coordinate system that is adjusted to the building’s face. The *X*-axis is parallel to the building facade, the *Y*-axis is vertical, and the *Z*-axis is perpendicular to the XY plane, see Figure 12.

**Figure 13:**
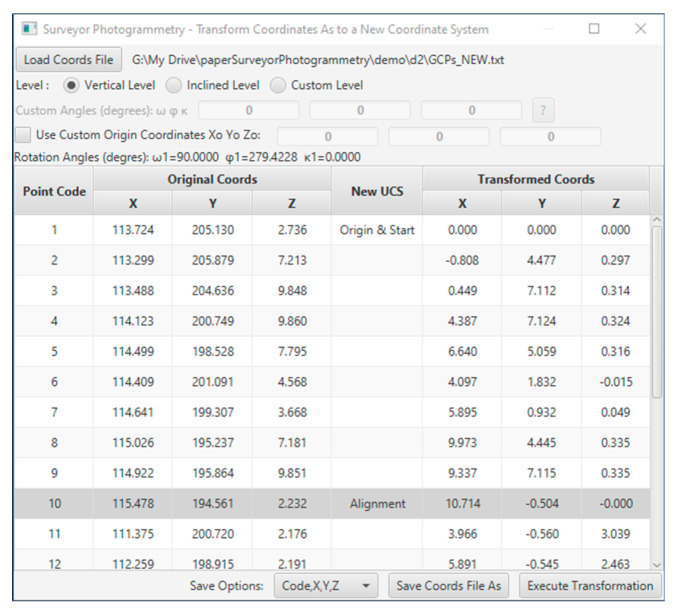
The coordinates transformation as to a user coordinate system on the Surveyor-Photogrammetry software version 6.0.

The coordinates transformation was performed in the Surveyor-Photogrammetry software version 6.0, see Figure 13. For the transformation, it is sufficient to define the direction of the building face with at least two points, one for the origin (Origin and Start) and one for the definition of the direction (Alignment).

The ground coordinates of the control and checkpoints, as to the new coordinate system, are listed in Table 11. Regarding the Z coordinates, their values range from −0.015 m to 3.039 m ensuring a satisfactory range of depth of field, theoretically ensuring reliable values for the external orientation of the photographs that will lead to reliable photogrammetric intersections.

Using this custom coordinate system, the accuracy in calculating the checkpoints in actual 3D measurements will be better estimated, especially when the *Z*-axis is in the same direction as the camera’s optical axis. The accuracy estimate along the *Z*-axis is critical in photogrammetry as it is the most sensitive, in terms of the accuracy of the measurements along this direction.

**Figure 14:**
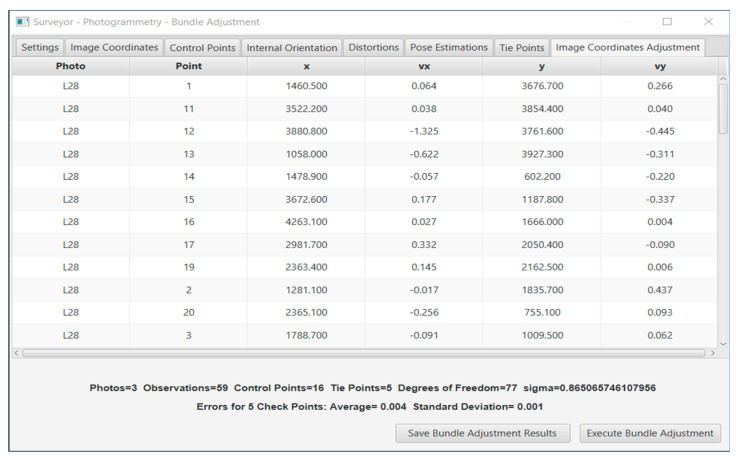
The bundle adjustment solution on the Surveyor-Photogrammetry software version 6.0.

To test the accuracy that can be achieved in the calculation of coordinates on the object, a photogrammetric survey on a building face was conducted. Specifically, the photogrammetric bundle adjustment method with additional parameters was used in the Surveyor-Photogrammetry environment, see an instance in Figure 14. The method accepts as input, the image, and the ground coordinates of the control points and estimates the coordinates of the checkpoints. Also, if selected, the internal orientation elements, i.e., the focal length, primary point coordinates, and distortion coefficients can be estimated too. 

The bundle adjustment method was performed for the still camera and the smartphone. Two solutions were extracted; in the first, the focal distance and the coordinates of the primary point were included as unknown parameters, and in the second, the distortion parameters *k*_1_, *k*_2_, *k*_3_, *p*_1_, *p*_2_ were added, as unknowns to the first case. The results of both cases are shown in Table 12 and Table 13, respectively.

The differences in the checkpoint coordinates, calculated using the two mapping methods, i.e., photogrammetry and topography, are an estimation of accuracy achieved. It is evident from Table 12 and Table 13 that the still camera outperforms the smartphone camera in all cases of various focal lengths. The accuracy differences between the two cameras are 10–17 mm. These accuracies determine the scales of the mapping products that can be produced photogrammetrically with this equipment. Furthermore, it significantly improves accuracy when distortion coefficients are considered.

### 3.3. Image Quality Test between the Smartphone and the Still Camera

**Figure 15:**
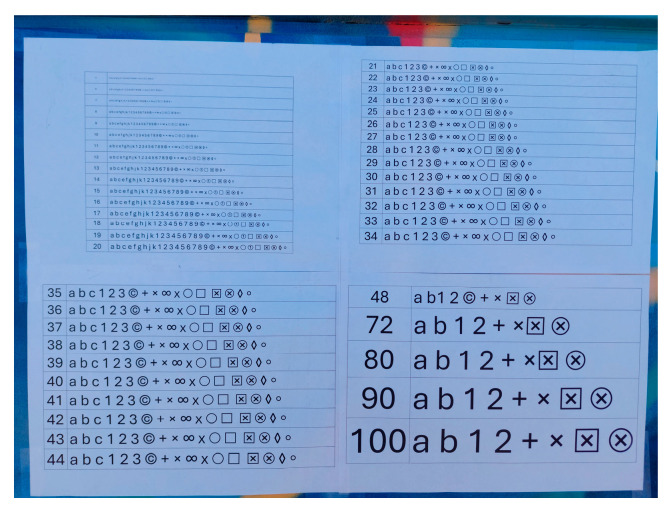
The board for the quality image test.

To compare the quality of the images from the smartphone camera and the still camera, a series of photographs was taken, where a board with printed alphanumeric characters and special symbols, in varying sizes, was photographed, see Figure 15.

**Figure 16:**
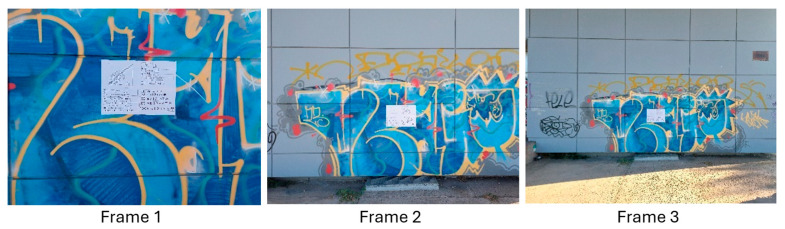
The 3 frames used for the image quality test—photos by the Samsung smartphone.

The experiment was divided into three sections, wherein in each section a specific horizontal width distance is photographically covered on the surface of a building, regardless of the shooting distance. These three sections were named frame 1, frame 2, and frame 3. For the still camera, the nominal focal lengths of 28, 34, 50, and 70 mm were used, while for the smartphone camera, the only available focal length of 5 mm was used. Therefore, in each frame, there are five photos, one from the smartphone camera and four from the still camera. The three photo frames, taken by the Samsung smartphone, are shown in Figure 16, for example.

During the experiment, all the photographs were taken in a brief period. In this way, the sun’s position, which affects the lighting, did not play a significant role in the quality of the photos. For the same reason, the humidity, pressure, and atmosphere composition remained the same during the photography and did not affect the quality of the photos. The same horizontal coverage on the object, in the frame photos, was achieved using as a guide the distinctive vertical lines on the facade of the building, see Figure 16, where the quality board was placed. At the same time, the camera was placed each time, along a line perpendicular to the facade of the building, which passed through the quality board. This way, the same horizontal coverage on the object and the same shooting angles were ensured.

Image quality is a subjective concept that depends on the purpose for which the image will be utilized and the viewer’s preferences. However, some quantitative metrics [30] can be used to measure and compare image quality, such as sharpness, contrast, noise, color accuracy, and dynamic range. Photogrammetry is mainly interested in identifying the edges of shapes in images. In this case, it was chosen to photograph alphanumeric characters and symbols in each frame. Depending on whether these are recognizable, distorted, or sharp, it will provide a clear picture of each camera’s image quality. In this way, the performance of the camera lens at different nominal focal lengths will also be studied. 

Selected parts of the photos where the board is depicted have been cropped from the frames and are presented in Table 14, Table 15 and Table 16.

The photos of the alphanumeric characters and symbols show that between the different nominal focal lengths of 28, 34, 50, and 70 mm, there are no significant differences in the quality of the images. This means that the performance of the lens used by the Sony still camera is satisfactory.

Comparing the photos from the Sony camera with those from the Samsung smartphone in Table 14, Table 15 and Table 16, there is a qualitative difference in the recognition, and the sharpness of the depicted characters and symbols, especially as the shooting distance increases. The results for Sony are slightly better, but in no case are there any big differences. A slightly larger difference in image quality is shown in frame 3, which corresponds to the longest shooting distance of the test.

At longer shooting distances, no comparison can be made, as the smartphone cannot use a longer focal length lens. The digital zoom capability, on the one hand, cannot be used in photogrammetry as it is not a central projection, and on the other hand, it has no practical effect as it does not provide additional information as it simply magnifies the original information from the imaging sensor. The length x height ratio of the still camera sensor is 6000 × 4000, i.e., 1.5:1, while the smartphone camera is 9248 × 6936, i.e., 1.33:1. Since the same horizontal area is photographed, the pixel ratio between the smartphone camera and the still camera is 6000:9248, which is 0.65 in favor of the smartphone sensor.

**Figure 17:**
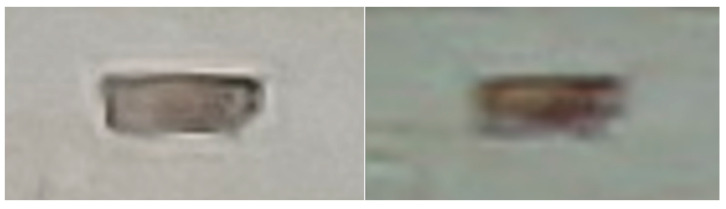
Construction detail. (**Left**): Samsung smartphone (zoom: 1310%) and (**Right**): Sony camera (zoom: 1919%).

Figure 17 is an additional sample related to the comparison of the two cameras’ image quality, depicting a construction detail on the face of a building.

Figure 17 shows differences between the two photographs, yet the outline and boundaries of the construction detail are evident in both cases. 

Some practical issues encountered and dealt with during photography, which may affect the quality of the images, are worth mentioning. 

**Figure 18:**
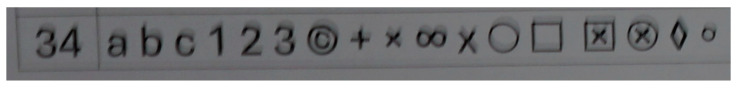
Quality image test for the Sony-28 mm no photo tripod used.

**Figure 19:**
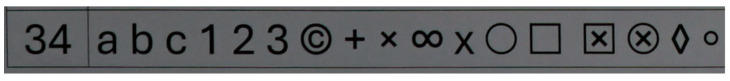
Quality image test for the Sony-28 mm photo tripod used.

The relative movement of the imaging sensor about the camera body creates issues in photogrammetric procedures, where the assumption is to maintain a constant geometry in the internal orientation in all shots. For this reason, the automatic image stabilization function in the Sony camera was disabled. To determine the effects of this setting, test shots were taken with and without a photo tripod, and the results are shown in Figure 18 and Figure 19, respectively.

With the automatic image stabilization function of the camera disabled, especially in the cases of poor lighting, where the shutter speed is slow, the photography results are “shaken”, see Figure 18. In the case that a photo tripod is used, and the time-delay function of the photo shot is parallel, the results are very satisfactory, see Figure 19. In the case of the smartphone, the problem of “shaken” photos did not occur.

Another issue noticed is that while both cameras’ shooting parameters are set to AUTO, the mobile phone camera achieves brighter photos than the professional still camera. This would have been different if the photo capture parameters were set to manual.

## 4. Discussion and Conclusions

The full-frame mirrorless camera has smoother distortion than the smartphone camera. Specifically, in a circular sector having as central the primary point, and as radius the sensor’s height, the distortion was low. Especially with the use of the 34 mm lens, where the distortion was limited to 5 pixels, while in this section, it did not exceed 11 pixels, regardless of the focal length. Outside this sector, significant distortions, of the order of 40 pixels, were observed particularly at the edges of the image. If the project requires high precision, the measurements should be limited to this area and the edges of the image should be avoided. Also, in the general case, there was no systematic symmetry in the distortions, around the primary point, indicating significant tangential distortions. In the smartphone, the distortions are more significant and change sharply. The correction of distortions brings a 41.9% improvement in the image, compared to 15.9–39.1% of the still camera. In addition, the distortion correction performance on the image surface appears smoother and more symmetrical compared to the still camera.

In terms of the accuracy in calculating coordinates in actual 3D measurements, it was found that the full-frame mirrorless camera achieves about four times more accurate results than smartphones. Specifically, 6 mm against 23 mm of the smartphone’s camera, when the distortions were not considered, corresponding to cartographic products at scales of 1:30 and 1:115. In the other case, when distortions are considered, the accuracy is 4 mm for the full-frame mirrorless camera against 14 mm for the smartphone camera, which corresponds to cartographic products at scales of 1:20 and 1:70, respectively. The distortion corrections led to an improvement in coordinates calculation accuracy of up to 39%.

Regarding image quality, it was found that, in conditions of sufficient lighting, and at a shooting distance of 3–15 m, which correspond to realistic distances of terrestrial photogrammetry applications, there is a slight lead in the full-frame mirrorless camera over the smartphone, especially to the longest shooting distance of the test. There can be no comparison at longer distances, due to the fixed lens of the smartphone’s camera.

The study answers the question of how comparable photo quality results can be achieved between a professional camera and a smartphone. The answer lies in the balance of performance, where there are more pixels on the one hand and a smaller number but a larger physical size and better camera lens quality on the other hand. In this case, the mobile phone has a 64 MP imaging sensor, while the still camera is 24.3 MP. In terms of lenses, the mobile phone has a 5 mm fixed-focus wide-angle lens, while the camera has an interchangeable focal length lens ranging from 28 mm to 70 mm. The significant difference between the two cameras, when used for actual 3D measurements, is due to the quality of the lenses and the smaller distortions that this brings. A small accuracy contribution is also made by slightly better-quality images, resulting in a more accurate selection of image coordinates.

For the full-frame mirrorless camera, the automatic image stabilization function was disabled because, in photogrammetry, the internal geometry of the camera must be constant during all shots. For this reason, a photo tripod should be used, and the shots should preferably be taken with a few seconds’ delay.

In general, regarding the use of the two cameras in photogrammetry applications, the full-frame mirrorless camera excels in accuracy, while the smartphone camera is a particularly good choice for situations where a quick solution is required, for close shooting distances, and with low accuracy requirements.

## Figures and Tables

**Table 1:** Cameras technical specifications.

	Sony Camera	Samsung Smartphone
Lens focal length (mm)	28–70	5
Sensor resolution (pixels)	6000 × 4000 (24.3 MP)	9248 × 6936 (64 MP)
Pixel size (mm)	0.005967	0.000800
Physical dimensions of the sensor (mm)	35.800 × 23.867	7.398 × 5.549

**Table 2:** The cameras and their output field of view.

	Sony-28 mm	Sony-34 mm	Sony-50 mm	Sony-70 mm	Samsung Smartphone
Output field of view along the horizontal sensor axis (deg)	63.219	52.391	36.611	26.729	68.086
Output field of view along the vertical sensor axis (deg)	44.305	36.043	24.691	17.847	53.835

**Table 3:** The re-projection error for calibrating cameras using OpenCV calibration.

Camera	Re-Projection Error
Sony-28 mm	3.205
Sony-34 mm	3.039
Sony-50 mm	3.542
Sony-70 mm	4.031
Samsung smartphone	5.206

**Table 4:** The intrinsic matrix for all cameras.

	Sony-28 mm	Sony-34 mm	Sony-50 mm	Sony-70 mm	Samsung Smartphone
*fx* (pixels)	4874.5	6097.9	9067.5	12,627.0	6844.1
*fy* (pixels)	4912.4	6147.5	9137.2	12,737.2	6830.6
*c_x_* (pixels)	2974.6	2981.6	2912.2	2916.4	4659.0
*c_y_* (pixels)	1975.8	1968.3	1916.2	2030.4	3485.1
*AspectRatio* (*fy*/*fx*)	1.008	1.008	1.008	1.009	0.998
Nominal *f* (mm)	28	34	50	70	5
Estimated *c* (mm)	29.085	36.384	54.103	75.341	5.475
*x_o_* (pixels)	−25.4	−18.4	−87.8	−83.6	35.0
*y_o_* (pixels)	24.2	31.6	83.7	−30.4	−17.0
*x_o_* (mm)	0.151	−0.110	−0.524	−0.499	0.028
*y_o_* (mm)	0.144	0.189	0.500	−0.181	−0.014

**Table 5:** Distortion coefficient parameters for all cameras.

	Sony-28 mm	Sony-34 mm	Sony-50 mm	Sony-70 mm	Samsung Smartphone
*k*_1_	–0.02604127	0.00637290	–0.02729633	–0.05253937	0.11334916
*k*_2_	0.09946586	0.13491890	−0.06231847	−0.88042237	−0.35944055
*k*_3_	−0.10377144	−0.32155154	0.89514366	11.18174144	0.32451562
*p*_1_	−0.00171816	−0.00077366	−0.00262152	0.00419812	0.00071273
*p*_2_	−0.00335183	−0.00036767	−0.00642417	−0.00171302	−0.00043841

**Table 6:** General elements of the bundle adjustment solution with additional parameters.

	Sony Camera	Samsung Smartphone
Photos	7	5
Control points	1813	1813
Observations	12,691	9065
Additional parameters	8	8
Degrees of freedom	25,332	18,092

**Table 7:** Cameras calibration using bundle adjustment with additional parameters.

	Sony-28 mm	Sony-34 mm	Sony-50 mm	Sony-70 mm	Samsung Smartphone
*sigma*	4.247	4.402	3.998	4.067	3.883
*c* (StDev) (pixels)	4873.2 (2.0)	6128.5 (3.7)	9061.5 (4.4)	12,587.4 (7.8)	6837.8 (1.6)
*x_o_* (StDev) (pixels)	−14.2 (2.0)	−14.5 (3.0)	−133.4 (5.8)	−149.0 (11.0)	−46.6 (1.7)
*y_o_* (StDev) (pixels)	28.31 (1.6)	14.61 (2.4)	117.38 (4.5)	−194.9 (8.3)	−10.0 (1.4)

**Table 8:** Color gradients for the error differences.

Error Differences	Color Gradients
error > 0.3 mm	
0.2 < error < 0.3	
0.1 < error < 0.2	
0 < error < 0.1	
−0.1 < error < 0	
−0.2 < error < −0.1	
error < −0.2	

**Table 9:** Comparison of evaluation images when using OpenCV and bundle adjustment with additional parameters.

Camera	OpenCV	Bundle Adjustment with Additional Parameters
Sony-28 mm	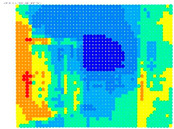	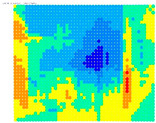
Sony-34 mm	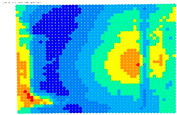	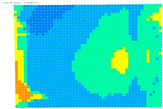
Sony-50 mm	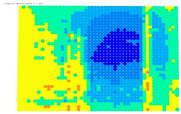	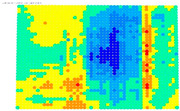
Sony-70 mm	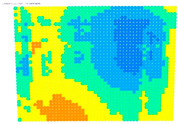	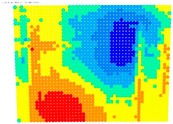
Samsung smartphone	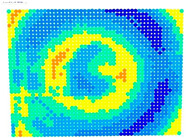	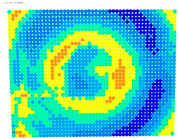

**Table 10:** Evaluation results for the cameras using the “Rect” indicator.

Camera	OpenCV Function	Bundle Adjustment
Sony-28 mm	+38.7%	+24.9%
Sony-34 mm	+34.7%	+27.2%
Sony-50 mm	+39.1%	+20.5%
Sony-70 mm	+15.9%	+4.3%
Samsung smartphone	+41.9%	+40.3%

**Table 11:** The ground coordinates of the control and checkpoints.

Code	X	Y	Z
1	0.000	0.000	0.000
2	−0.808	4.477	0.297
3	0.449	7.112	0.314
4	4.387	7.124	0.324
5	6.640	5.059	0.316
6	4.097	1.832	−0.015
7	5.895	0.932	0.049
8	9.973	4.445	0.335
9	9.337	7.115	0.335
10	10.714	−0.504	0.000
11	3.966	−0.560	3.039
12	5.891	−0.545	2.463
13	−0.928	−0.478	0.031
14	−0.503	8.073	0.322
15	8.093	8.505	0.337
16	11.483	7.180	0.351
17	4.605	4.624	0.310
18	12.152	0.159	0.019
19	2.335	3.996	0.303
20	2.303	8.499	0.330
21	5.007	0.311	0.972

**Table 12:** Bundle adjustment solution with auto-calibration for the focal length and the coordinates of the primary point.

	Sony-28 mm	Sony-34 mm	Sony-50 mm	Sony-70 mm	Samsung Smartphone
sigma	1.664	1.017	1.626	1.123	8.399
Observations	59	60	40	38	62
Control points	16	16	14	11	16
Checkpoints	5	5	4	3	5
Degrees of freedom	82	84	47	46	88
Average Z error (St Dev) (mm)	2 (2)	3 (2)	2 (2)	2 (2)	8 (9)
Average 2D error (St Dev) (mm)	5 (2)	2 (1)	2 (1)	5 (0)	22 (10)
Average 3D error (St Dev) (mm)	6 (2)	3 (2)	1 (3)	6 (1)	23 (10)

**Table 13:** Bundle adjustment solution with auto-calibration for the focal length, the coordinates of the primary point, and the distortion coefficients.

	Sony-28 mm	Sony-34 mm	Sony-50 mm	Sony-70 mm	Samsung Smartphone
sigma	0.874	0.997	1.463	0.503	4.251
Observations	59	60	40	38	62
Control points	16	16	14	11	16
Checkpoints	5	5	4	3	5
Degrees of freedom	77	79	42	41	83
Average Z error (St Dev) (mm)	2 (3)	3 (2)	2 (2)	1 (1)	10 (12)
Average 2D error (St Dev) (mm)	2 (1)	2 (1)	3 (1)	2 (1)	9 (4)
Average 3D error (St Dev) (mm)	4 (1)	3 (2)	4 (1)	3 (1)	14 (5)

**Table 14:** Comparative image quality samples—frame 1.

Sony-28 mm	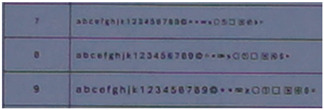
Sony-34 mm	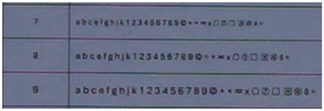
Sony-50 mm	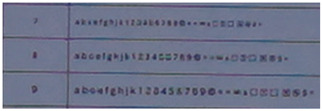
Sony-70 mm	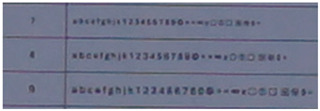
Samsung smartphone	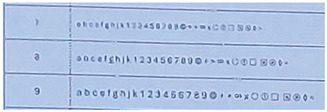

**Table 15:** Comparative image quality samples—frame 2.

Sony-28 mm	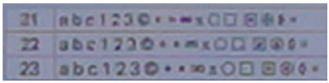
Sony-34 mm	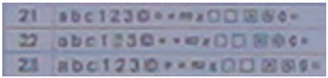
Sony-50 mm	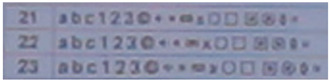
Sony-70 mm	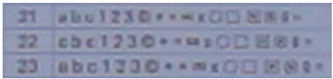
Samsung smartphone	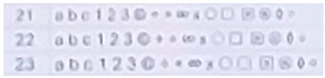

**Table 16:** Comparative image quality samples—frame 3.

Sony-28 mm	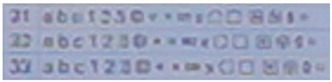
Sony-34 mm	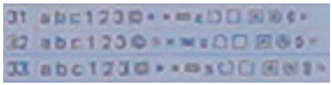
Sony-50 mm	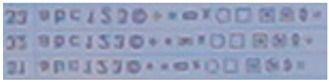
Sony-70 mm	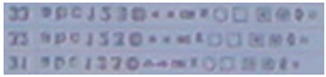
Samsung smartphone	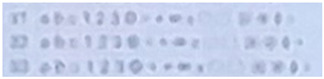

## Data Availability

Data are contained within the article.
